# A three-miRNA signature as promising non-invasive diagnostic marker for gastric cancer

**DOI:** 10.1186/s12943-015-0473-3

**Published:** 2015-11-25

**Authors:** Vivian Yvonne Shin, Enders K. O. Ng, Vivian W. Chan, Ava Kwong, Kent-Man Chu

**Affiliations:** Department of Surgery, Queen Mary Hospital, The University of Hong Kong, Pokfulam, Hong Kong, SAR China; Department of Surgery, Hong Kong Sanatorium & Hospital, Hong Kong, SAR China; Hong Kong Hereditary Breast Cancer Family Registry, Hong Kong, SAR China

**Keywords:** Circulating microRNA, Gastric cancer, Diagnostic marker

## Abstract

**Background:**

Despite the declining incidence of gastric cancer, mortality rate remains high due to late presentation. We aimed to evaluate the sensitivity of miRNA as a diagnostic marker for gastric cancer in the circulation.

**Methods:**

Plasma samples from 3 independent groups comprise 123 gastric cancer patients and 111 healthy controls for miRNA profiling from microarray screening.

**Results:**

Microarray data showed that 25 miRNAs were upregulated in gastric cancer patients and 6 highly expressed miRNAs (miR-18a, miR-140-5p, miR-199a-3p, miR-627, miR-629 and miR-652) were selected for validation. In an independent validation set, levels of miR-627, miR-629 and miR-652 were significantly higher in gastric cancer patients than healthy controls (*P* <0.0001). An algorithm with improved sensitivity and specificity as gastric cancer classifier was adopted and validated in another random set of 15 plasma samples. Results showed that combination of 3 miRNAs obtained the highest area under curve, with a cut-off at 0.373, with a sensitivity of 86.7 % and a specificity of 85.5 %.

**Conclusion:**

This study revealed a three-miRNA signature as a promising classifier for gastric cancer, and greatly enhances the feasibility of circulating miRNAs as non-invasive diagnostic marker for this disease.

**Electronic supplementary material:**

The online version of this article (doi:10.1186/s12943-015-0473-3) contains supplementary material, which is available to authorized users.

## Background

Gastric cancer is one of the leading causes of cancer mortality in the world, and it has a particularly high incidence in Asian countries including China. Despite the declining incidence of gastric cancer, there are still more than 1 million cases newly diagnosed and 850,000 deaths globally each year [1]. The mortality rate remains high due to late presentation, since early stage of gastric cancer is either asymptomatic or presents with non-specific symptoms. For advanced diseases, the overall 5-year survival following surgical resection is 30–40 %, as compared to 70–90 % in early stage [2]. To date, endoscopic and pathological examinations are the common techniques for cancer diagnosis. Despite their sensitivity and specificity to visualize and locate the site of malignancy, these approaches are invasive in nature which impede patients from routine screening for gastric cancer. Conventional serum tumour markers, namely, carcinoembryonic antigen (CEA) and carbohydrate antigen 19.9, however, are not tissue specific and expressed in most of the gastrointestinal cancers. Our group has shown that serum migration-inhibitory factor (MIF) had a better diagnostic value than CEA, however, combined serum MIF and CEA would have a better 5-year survival prognosis than individual marker [3]. Serum E-cadherin level was found to be positively correlated with disease recurrence, and this could be a better marker than CEA in predicting disease recurrence [4, 5]. Therefore, there is an urge to look for a non-invasive diagnostic biomarker that could be easily detected in serum, plasma or urine for early diagnosis for gastric cancer to greatly improve the mortality rate.

Many recent studies revealed that microRNAs (miRNAs) were actively involved in development, differentiation, inflammation, and pathogenesis of various malignancies. They belong to a class of small non-coding RNAs about 19–25 nucleotides in length, and are able to bind complementary sequences in 3′-untranslated regions (3′-UTR) of various target mRNAs to promote degradation or translational repression [6]. Studies have shown that over 30 % of human genes are regulated by miRNAs, in which a single miRNA controls over hundreds of RNA [7, 8]. MiRNAs also function as tumour suppressors or oncogenes in various types of cancer. Because miRNAs are very specific for different types of tissues and even for types of cells within those tissues, they are potentially useful for diagnosis, predicting clinical outcome or therapeutic targets in cancer patients. By comparing the miRNA expression profiles in tumour versus adjacent non-tumour tissues, distinct patterns of up- or down-regulation of miRNAs could be found in different types of cancers [9–12]. These cancer specific miRNAs expression patterns could be used for diagnosis or monitor the efficiency of follow-up treatment.

With the development of microarray platforms, researchers could easily differentiate the oncogenic or tumour suppressive miRNAs in various human malignancies. We have previously developed a robust protocol to profile miRNA expression in the circulation of colorectal cancer patients [12]. With the use of PCR-based miRNA array, we could profile the miRNA expression in plasma, as well as in paired tumour and adjacent non-tumour tissues. There are studies reporting that miRNA is more stable than mRNA in the circulation and yet specific miRNA is originate from tumour site [13, 14]. In this study, we compare the miRNA expression profile in plasma of gastric cancer patients with healthy controls. We further validated the levels of miRNAs in three independent sets of gastric cancer patients and associated with tumour progression. We proposed that circulating miRNA signature could act as a potential molecular marker for diagnosis and therapeutic targets for gastric cancer.

## Results

### Discovery of miRNA expression profile in the plasma of gastric cancer patients

**Fig. 1:**
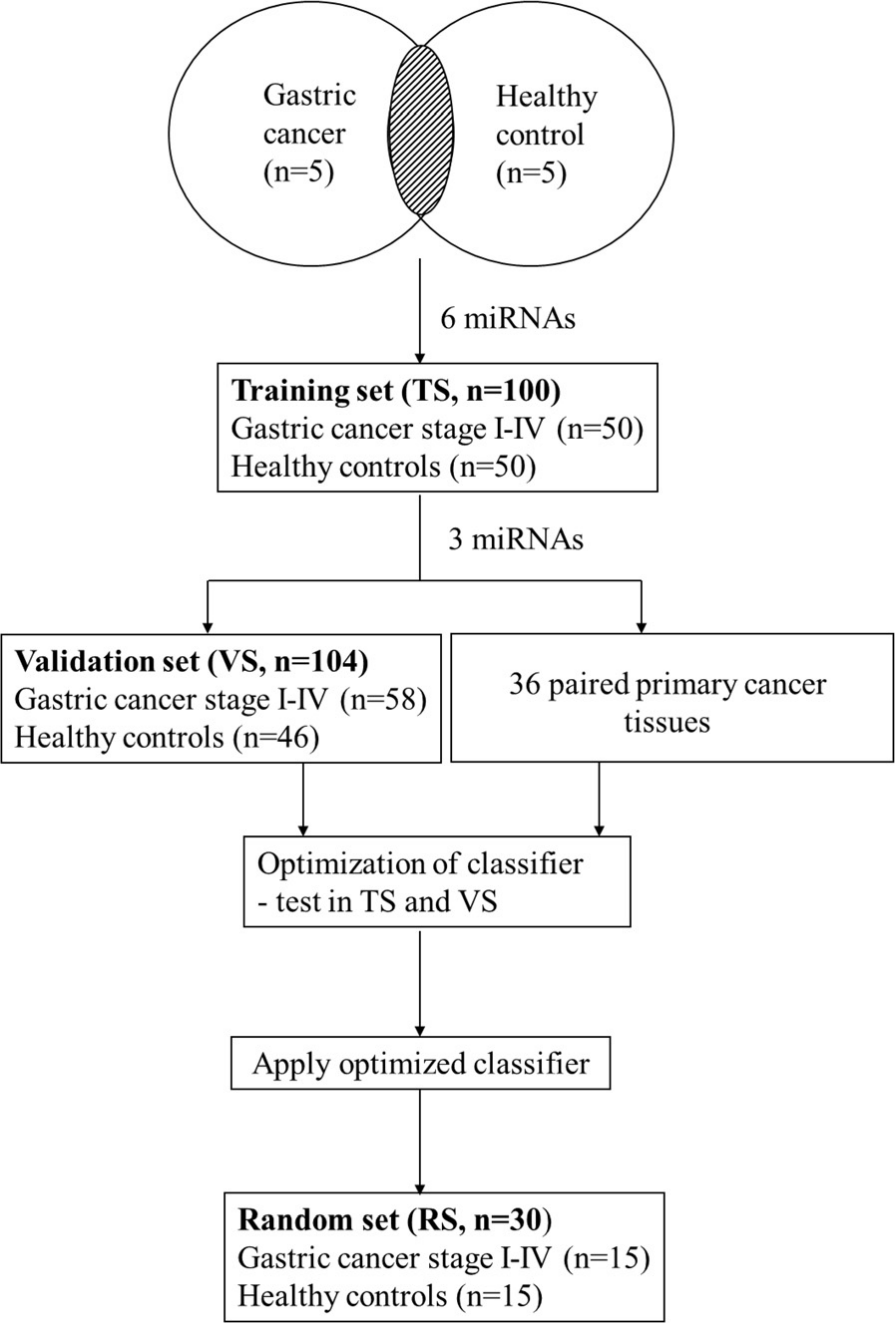
An overview of the workflow of the study design

It is speculated that miRNAs originate from gastric tumor will enter into the circulation, and could be potentially used as molecular marker for early detection of gastric cancer. An overview of the study design is illustrated in Fig. 1. To identify the miRNA expression profiles in gastric cancer, we first compared the miRNA expression in plasma of gastric cancer patients (*n* = 5) with healthy controls (*n* = 5) using microarray analysis. There were 77 (out of 377) miRNAs differentially expressed in gastric cancer, of which 25 were upregulated (Table 1) and 52 were downregulated (Additional file 1: Table S1), with a cut-off value of 2-fold difference.

**Table 1:** Upregulated miRNAs associated with gastric cancer at a cut-off value of 2-fold difference

MiRNAs	Fold change
has-miR-140-5p	5.48
has-miR-409-3p	5.20
has-miR-143	5.12
has-miR-199a-5p	4.08
has-miR-423-3p	3.81
has-miR-338-3p	3.53
has-miR-33a	3.38
has-miR-652	3.17
has-miR-551b	3.15
has-miR-18a	2.64
has-miR-584	2.55
has-miR-181d	2.53
has-miR-28-5p	2.48
has-miR-627	2.46
has-miR-376a	2.45
has-miR-503	2.40
has-miR-628-3p	2.39
has-miR-1	2.37
has-miR-629	2.34
miR-625-3p	2.18
has-miR-7	2.14
has-miR-590-5p	2.07
has-miR-199a-3p	2.06
has-miR-766	2.05
has-miR-335	2.02

### Selection of potential miRNAs as diagnostic markers

**Fig. 2:**
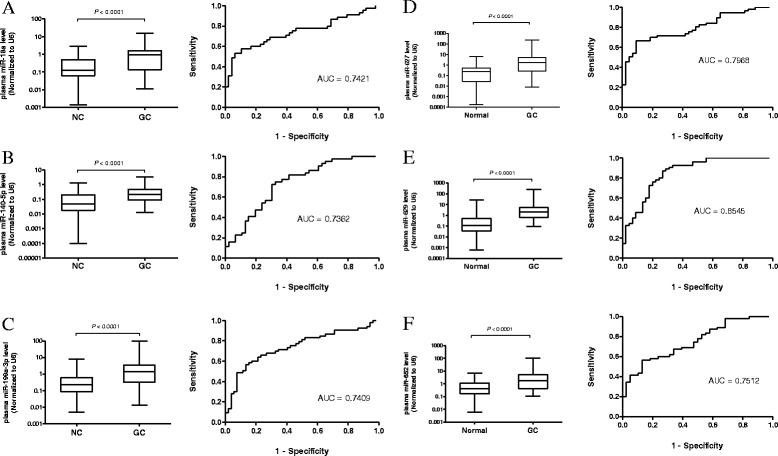
Validation of plasma (**a**) miR-18a, (**b**) miR-140-5p, (**c**) 199a-3p, (**d**) miR-627, (**e**) miR-629 and (**f**) miR-652 levels in training set (TS). Expression levels were normalized to U6. Box plots of six miRNAs in plasma of gastric cancer patients (*n* = 50) and healthy controls (*n* = 50) The boxes mark the interval between the 25^th^ and 75^th^ percentiles, and the lines inside the box denote the medians. The whiskers represent the interval between the 10^th^ and 90^th^ percentiles. Statistically significant differences were analyzed using Mann–Whitney test. Receiver-operating characteristic (ROC) curve analysis of miRNA for discriminating gastric cancer patients from healthy controls

We then carried out real-time qPCR to validate these 25 upregulated miRs in both plasma and paired tumor/non-tumor tissues samples from the same patients (*n* = 20) (data not shown). Next, we selected top 6 most upregulated miRs (miR-18a, miR-140-5p, 199a-3p, miR-627, miR-629 and mir-652) that significantly expressed in both plasma and tissues samples for the second part of the study. We then selected these 6 upregulated miRNAs for further validation using real-time qPCR analysis in an initial training set (TS) containing 50 gastric cancer cases of different stages (stages I, *n* = 8; stage II, *n* = 11; stage III, *n* = 15; stage IV, *n* = 16) and 50 age- and sex-matched healthy controls. The expression levels of miR-18a, miR-140-5p, 199a-3p, miR-627, miR-629 and miR-652 were comparable with the microarray data (Fig. 2a-f). Results showed that the fold change of these miRNAs initially screened by microarray were consistent with the qPCR analysis. The AUC of miR-627 (AUC = 0.7968), miR-629 (AUC = 0.8545) and miR-652 (AUC = 0.7512) were highest in this training set.

### Validation of miR-627, miR-629 and miR-652 as biomarkers to differentiate gastric cancer cases

**Fig. 3:**
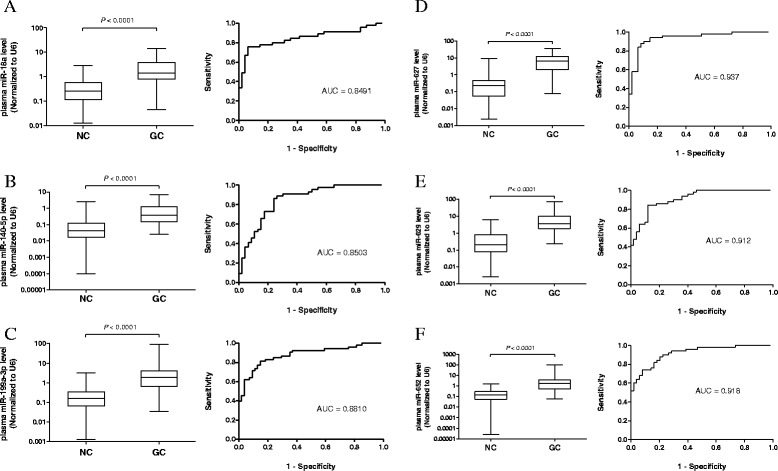
Selection and validation of (**a**) miR-18a, (**b**) miR-140-5p, (**c**) 199a-3p, (**d**) miR-627, (**e**) miR-629 and (**f**) miR-652 in an independent validation set (VS). Box plots and ROC curve analysis of miRNAs in the plasma of gastric cancer patients (*n* = 58) and healthy controls (*n* = 46). The boxes mark the interval between the 25^th^ and 75^th^ percentiles, and the lines inside the box denote the medians. The whiskers represent the interval between the 10^th^ and 90^th^ percentiles. Statistically significant differences were analyzed using Mann–Whitney test. Receiver-operating characteristic (ROC) curve analysis of miRNA for discriminating gastric cancer patients from healthy controls

We next evaluated the expression levels of miR-18a, miR-140-5p, 199a-3p, miR-627, miR-629 and miR-652 in an independent validation set (VS) consist of 58 gastric cancer cases of different stages (stages I, *n* = 8; stage II, *n* = 2; stage III, *n* = 26; stage IV, *n* = 22) and 46 age- and sex-matched healthy controls. Results showed that gastric cancer patients have a higher expression levels of miR-627, miR-629 and miR-652 relative to healthy controls (Fig. 3a-f), all of which were statistically significant (*P* < 0.0001).

**Fig. 4:**
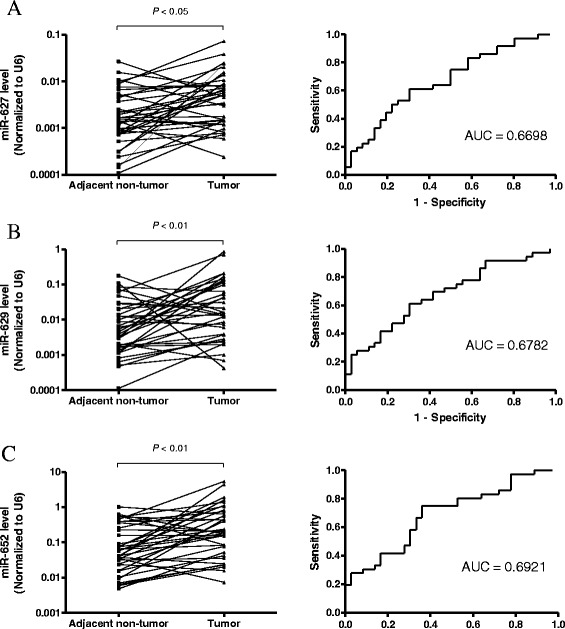
Expression levels of (**a**) miR-627, (**b**) miR-629 and (**c**) miR-652 in paired gastric tumor tissues and adjacent non-tumor counterparts (*n* = 36). Expression levels were normalized to U6. Statistically significant differences were analyzed using Wilcoxon test. Receiver-operating characteristic (ROC) curve analysis of miRNA for discriminating gastric cancer patients from healthy controls

Since miR-627, miR-629 and miR-652 had the highest AUC in both TS and VS, we further examined the expression levels in 36 paired primary cancer tissues from VS, by comparing the tumor tissues with its adjacent non-tumor counterparts. In consistent with the plasma data, tumor tissue levels of miR-627, miR-629 and miR-652 were significantly higher than in non-tumor counterparts (*P* < 0.05, <0.01 and <0.01 respectively). The ROC curves analysis of these miRNAs showed that the AUC were 0.6698, 0.6782 and 0.6921 accordingly (Fig. 4a-c).

### Optimization of miRNAs as potential gastric cancer classifiers

To improve the specificity of the classifier to differentiate gastric cancer cases with non-gastric cancer cases, we designed a new algorithm by combining miRNAs into classifier A (miR-627 and miR-629), classifier B (miR-627 and miR-652), classifier C (miR-629 and miR-652) and classifier D (miR-627, miR-629 and miR-652). To test whether these four classifiers could be more specific to detect gastric cancer cases, we tested these four classifiers in the TS and VS respectively. By applying classifier D to TS, it could use to distinguish gastric cancer cases from healthy controls and the AUC increased to 0.902 (Table 2). The sensitivity was 0.855 and the specificity was 0.757. And we observed a high significant different between gastric cancer cases and healthy controls (*P* < 0.0001, *t*-test). Similarly, the same classifier apply to VS, the AUC was determined to be 0.969. The probability scores were markedly different between gastric cancer cases and healthy controls (*P* < 0.001, *t*-test). We observed a sensitivity of 0.920 and specificity was 0.935. We observed that all these classifiers have improved sensitivity and specificity than individual miRNA, in which classifier D obtained the highest AUC among all the classifiers.

**Table 2:** ROC curve analyses of the potential classifiers

	TS				VS			
Classifier	Sensitivity	Specificity	AUC	95 % CI	Sensitivity	Specificity	AUC	95 % CI
Classifier A (miR-627 & miR-629)	0.804	0.773	0.878	0.814–0.942	0.9	0.894	0.948	0.905–0.990
Classifier B (miR-627 & miR-652)	0.821	0.757	0.842	0.763–0.922	0.88	0.935	0.965	0.931–0.991
Classifier C (miR-629 & miR-652)	0.800	0.821	0.884	0.817–0.952	0.960	0.837	0.963	0.932–0.995
Classifier D (miR-627, miR-629 & miR-652)	0.855	0.757	0.902	0.842–0.962	0.92	0.935	0.969	0.938–1.001

**Fig. 5:**
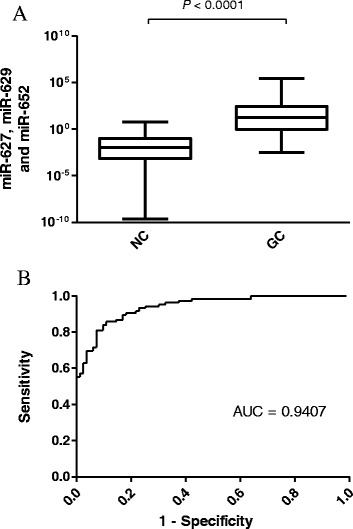
Validation of gastric cancer classifier in different validation sets. **a** Box plot and ROC curve analysis of combining 3 miRNAs in random set (RS) with gastric cancer patients (*n* = 15) and healthy controls (*n* = 15). **b** Box plot and ROC curve analysis of combining 3 miRNAs in TSVS with gastric cancer patients (*n* = 108) and healthy controls (*n* = 96). The boxes mark the interval between the 25^th^ and 75^th^ percentiles, and the lines inside the box denote the medians. The whiskers represent the interval between the 10^th^ and 90^th^ percentiles. Statistically significant differences were analyzed using Mann–Whitney test

In this setting, we further examined the efficacy based on classifier D in the random set (RS) containing 15 gastric cancer cases of different stages (stages I, *n* = 4; stage II, *n* = 2; stage III, *n* = 5; stage IV, *n* = 4) and healthy controls. Gastric cancer cases were significantly differentiated with healthy controls by more than 10-folds, the AUC was determined to be 0.941 (*P* < 0.0001; Fig. 5a). In addition, if we combined the datasets of TS and VS, the optimal cut-off value of classifier D was at 0.373 with a sensitivity of 86.7 % and a specificity of 85.5 %, and the AUC was 0.941 (Fig. 5b).

### Association with progression of gastric cancer

**Fig. 6:**
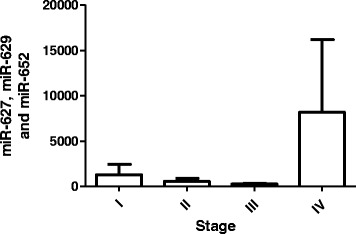
Correlation between gastric cancer classifier and tumor stage in plasma of gastric cancer patients (*n* = 123)

To examine whether the gastric cancer classifier is correlated with pathological stage, we analyze the relative expression of classifier D with the TNM stage in combined datasets of TS and VS. Data showed that there is no direct correlation between the expression of classifier D and the TNM stage (Fig. 6), this implicate miRNA expression is a stage-independent diagnostic marker in patients with gastric cancer.

## Discussion

Gastroscopy remains the gold standard for detection of gastric cancer, which usually has a high false negative rate, due to the lack of noticeable symptoms of gastric cancer. Therefore, patients usually diagnosed with advanced cancer when underwent these examinations. Hence, the development of gastric cancer screening tool would effectively reduce the overall mortality. To date, there is no reliable non-invasive blood based classifier for the detection of gastric cancer.

Several studies reported that miRNA pattern is distinct in different cancer types. MiR-21 is one of the miRNAs that is known to play a role in various types of carcinomas [15, 16], including gastric carcinoma [17]. Studies have shown that miR-21 is known to increase in gastric cancer, and associated with tumor cell growth and invasion [18, 19]. However, it is not suitable to be a tissue-specific diagnostic marker of gastric cancer, due to its high expression in most of the cancerous tissues. This prompted us to discover the feasibility of other miRNAs in plasma for diagnosis.

In this study, we used a miRNA microarray platform to screen the differentially expressed miRNAs in plasma of gastric cancer patients, and several upregulated miRNAs (miR-18a, miR-140-5p, 199a-3p, miR-627, miR-629 and mir-652) were selected for validation. We then selected the three miRNAs (miR-627, miR-629 and miR-652) with highest ROC curve and performed a large validation study on the expression levels. In both training (*n* = 50) and validation sets (*n* = 58), these 3 miRNAs were highly expressed in gastric cancer cases when compared with healthy controls. The algorithm we used to optimize the gastric cancer classifier (by multiplication of two highest expression levels among 3 miRNAs), which can be used as a biomarker to discriminate gastric cancer patients with healthy controls. The classifier was validated again in TS and VS, and AUC were determined to be 0.902 and 0.969 respectively with improved sensitivity and specificity when compared to individual miRNA, implicating that this classifier is robust biomarker for gastric cancer. We further validated the classifier in a RS (mixture of gastric cancer and healthy controls samples), and could discriminate gastric cancer patients from healthy controls with AUC calculated to be 0.942. There is no association between the expression levels of the classifier (or individual miRNAs) and TNM stage in plasma of gastric cancer patients. It is widely accepted that miRNA is being released into the circulation from the primary gastric cancer site, therefore it is expected to detect a similar trend of elevation of miRNA expression in gastric cancer tissues. A consistent higher expression levels of miR-627, miR-629 and miR-652 were detected in tumor tissues than adjacent non-tumor counterparts (*n* = 36) (Fig. 3b), which echoed with the findings in plasma of gastric cancer patients.

To the best of our knowledge, this is the first study reported that expression of plasma miR-627 is more than 10-fold higher in gastric cancer patients than in healthy controls. It is worthwhile to investigate the specificity of this miRNA by examining its expression in other cancerous tissues. High level of miR-629* was detected in the plasma of xenograft mice which is originated from human prostate cancer xenograft [14]. This study provide evidence that tumor-derived miRNA could be detected in the circulation. Several studies reported that mir-652 is associated with tumor progression of osteosarcoma [20] and hepatocellular carcinoma [21]. Another group reported that deregulation of miR-652 was identified as a biomarker for schizophrenia [22]. However, in primary squamous cell lung carcinoma tissues, the levels of miR-652 were downregulated as compared with normal counterparts [23]. These studies revealed that the function of miRNA is different in various carcinomas and yet further study mechanistic on the oncogenic pathways may help to understand the progression of the disease.

## Conclusion

In conclusion, our data demonstrated that circulating miRNAs could be a sensitive biomarker for diagnosis of gastric cancer. This study revealed a new algorithm to discriminate gastric cancer cases and non-gastric cancer cases, the classifier (by combining 3 miRNAs) illustrated a promising discrimination of gastric cancer cases in different validation sets, which is far more sensitive than conventional tumor marker (eg CEA and CA 19.9). Further studies are warranted to examine the expression levels of selected miRNAs after surgery to verify the usefulness of the classifier to predict recurrence or therapeutic strategy during follow-up. This study may open up new opportunity to develop an economical non-invasive diagnostic tool for early detection of gastric cancer to reduce mortality.

## Methods

### Clinical samples

Pre-resection plasma and respective paired primary tumor/adjacent non-tumor tissue samples were obtained at Queen Mary Hospital between 2006 to 2010, while healthy volunteers were recruited at the Hong Kong Sanatorium and Hospital from 2009–2010. To discover and validate a specific biomarker for gastric cancer, this study consists of 3 independent sets of cases and controls. The training set (TS) contained 50 gastric cancer cases from 2006–2007, validation set (VS) contain 58 gastric cancer cases from 2008–2010 and random set contain 15 gastric cancer cases from 2008–2010, each independent set includes stage I to IV patients.

Plasma and tissue samples are stored at the frozen tissue bank of the Department. The collection and storage of such tissue samples have been approved by the Institutional Review Board. Informed consent has been obtained from each patient. Characteristics of patients such as gender, age, co-morbidity, presenting symptoms and signs, operative findings and staging will be prospectively collected into our standard electronic database. Clinical characteristics of gastric cancer patients were summarized in Table 3. These gastric plasma/tissues will be subjected to miRNA profiling and correlated with the clinicopathological factors in gastric cancer patients.

**Table 3:** Clinical characteristics of gastric cancer patient

	Training set (*n* = 50)	Validation set (*n* = 58)	Random set (*n* = 15)	*P* value	Total (*n* = 123)
Age [years; mean (SD)]	62.8 (18.3)	67.2 (16.4)	67.1 (13.4)	0.366	64.40 (16.88)
Sex				0.499	
Men	31	33	11		75/123 (61 %)
Women	19	25	4		48/123 (39 %)
Depth of invasion (T)				0.932	
T1	7	4	2		13/123 (11 %)
T2	12	13	3		28/123 (23 %)
T3	18	25	6		49/123 (40 %)
T4	13	16	4		33/123 (27 %)
Lymph-node metastasis				0.361	
(N)	13	10	4		27/123 (22 %)
N0	10	8	2		20/123 (16 %)
N1	26	40	8		74/123 (60 %)
N2	1	0	1		2/123 (2 %)
N3					
Distant metastasis				0.554	
No	37	46	13		96/123 (78 %)
Yes	13	12	2		27/123 (22 %)
Stage				0.088	
I	8	8	4		20/123 (16 %)
II	11	2	2		15/123 (12 %)
III	15	26	5		46/123 (38 %)
IV	16	22	4		42/123 (34 %)

### MiRNA extraction

Total RNA containing small RNA was extracted from 500 μl of plasma using Trizol LS reagent (Invitrogen, Carlsbad, California, USA) and miRNeasy Mini Kit (Qiagen, Hilden, Germany) according to the manufacturer’s protocol. After phase separation by chloroform addition and centrifugation, 1.5 volumes of 100 % ethanol was added to the aqueous phase and the mixture was loaded into miRNeasy column (Qiagen). DNase treatment (Qiagen) was carried out to remove any contaminating DNA. The final elution volume was 30 μl. The concentrations of all RNA samples were quantified by NanoDrop 1000 (Nanodrop, Wilmington, Delaware, USA).

### MiRNA microarray

In the screening phase, we profiled 10 age- and sex- matched individuals (5 gastric cancer patients vs 5 normal controls) using a miRCURY LNA Array (Exiqon) which contained 730 human miRNAs. This system is a real-time PCR-based array containing a panel of 384 well-established mature miRNA assays. The kit contains all reagents and primers, reverse transcription and qPCR. In brief, a poly-A tail is added to the mature miRNA template and then synthesized to cDNA by a poly-T primer with a 3′ degenerate anchor and 5′ universal tag. The cDNA is amplified by miRNA-specific and LNA™ -enhanced forward and reverse primers. SYBR Green PCR will be performed in LC480 Real-time PCR system (Roche).

### MiRNA validation by real-time quantitative RT-PCR

Plasma/tissues RNA containing miRNA is reverse transcribed to cDNA using miScript Reverse Transcription kit (Qiagen) according to the manufacturer’s instructions. qPCR is performed using SYBR real-time PCR using miScript SYBR Green PCR kit (Qiagen) with the manufacturer provided miScript Universal primer and the miRNA-specific forward primers in ABI 7900 Real-time PCR system (Applied Biosystems). The miRNA-specific primer sequences are designed by us based on the miRNA sequences obtained from the miRBase database (release 19). Each sample is run in duplicates and the expression levels of miRNAs are normalized to an endogenous control RNU6B (U6). Fold change in expression of each gene is calculated by a comparative threshold cycle (C_t_) method using the formula: 2^-[ΔCt(tumor)- ΔCt(control)]^.

### Statistical analysis

The significance of plasma miRNA levels was determined by the Mann–Whitney test, Wilcoxon test, *t*-test or Kruskal–Wallis test where appropriate. The Spearman rank order correlation test was used to examine correlation relationships between the levels of the miRNA markers. Multivariate logistic regression model will be established and leave-one-out cross validation will be performed to find the logistic model. Receiver operating characteristic (ROC) curves were established for discriminating patients with or without gastric cancer. All *P*-values are two-sided and a value less than 0.05 were considered statistically significant. All statistical calculations were performed by the SPSS software (version 17.0).

## Additional file

Additional file 1: Table S1. Downregulated miRNAs associated with gastric cancer at a cut-off value of 2-fold difference. (DOCX 16 kb)
