# Supplementary material for: A three-miRNA signature as promising non-invasive diagnostic marker for gastric cancer
Source: Mol Cancer. 2015 Nov 25;14:202. doi: 10.1186/s12943-015-0473-3 (PMC4659169; doi:10.1186/s12943-015-0473-3)
Supplement: Additional file 1: Table S1. — Downregulated miRNAs associated with gastric cancer at a cut-off value of 2-fold difference. (DOCX 16 kb) [file 12943_2015_473_MOESM1_ESM.docx]

Supplementary Table 1. Downregulated miRNAs associated with gastric cancer at a cut-off value of 2-fold difference

| Downregulated miRNAs | Fold change | Downregulated miRNAs | Fold change |
| --- | --- | --- | --- |
| hsa-miR-126 | 0.49 | hsa-miR-211 | 0.35 |
| hsa-miR-16 | 0.49 | hsa-miR-200a | 0.34 |
| hsa-miR-148a | 0.48 | hsa-miR-194 | 0.34 |
| hsa-miR-30b* | 0.48 | hsa-miR-10a | 0.33 |
| hsa-miR-490-3p | 0.47 | hsa-miR-342-3p | 0.32 |
| hsa-miR-493 | 0.45 | hsa-miR-181a | 0.32 |
| hsa-miR-222 | 0.44 | hsa-miR-195 | 0.32 |
| hsa-miR-193b | 0.43 | hsa-miR-99a | 0.31 |
| hsa-miR-205 | 0.43 | hsa-miR-663 | 0.29 |
| hsa-miR-99b | 0.42 | hsa-miR-622 | 0.28 |
| hsa-miR-346 | 0.41 | hsa-miR-134 | 0.28 |
| hsa-miR-145 | 0.41 | hsa-miR-150 | 0.26 |
| hsa-miR-665 | 0.41 | hsa-miR-124 | 0.24 |
| hsa-miR-92a | 0.41 | hsa-miR-125a-5p | 0.24 |
| hsa-miR-377 | 0.40 | hsa-miR-125b | 0.23 |
| hsa-miR-26a | 0.40 | hsa-miR-1979 | 0.23 |
| hsa-miR-30b | 0.40 | hsa-miR-934 | 0.23 |
| hsa-miR-631 | 0.39 | hsa-miR-10b | 0.23 |
| hsa-miR-192 | 0.37 | hsa-miR-373* | 0.23 |
| hsa-miR-30c | 0.37 | hsa-miR-765 | 0.22 |
| hsa-miR-452 | 0.36 | hsa-miR-502-5p | 0.18 |
| hsa-let-7c | 0.36 | hsa-miR-122 | 0.15 |
| hsa-miR-30a | 0.36 | hsa-miR-885-5p | 0.14 |
| hsa-miR-888 | 0.36 | hsa-miR-940 | 0.12 |
| hsa-miR-887 | 0.36 | hsa-miR-483-3p | 0.09 |
| hsa-miR-886-3p | 0.36 | hsa-miR-375 | 0.06 |
